# Formation and Applications of Typical Basic Protein-Based Heteroprotein Complex Coacervations

**DOI:** 10.3390/foods13203281

**Published:** 2024-10-16

**Authors:** Yufeng Xie, Qingchen Liu, Yubo Ge, Yongqi Liu, Rui Yang

**Affiliations:** 1 College of Food Science and Engineering, Harbin University, Harbin 150086, China; 2 Tianjin Key Laboratory of Food Quality and Health, Tianjin University of Science and Technology, Tianjin 300457, China

**Keywords:** heteroproteins, basic protein, complex coacervation, protein interactions

## Abstract

Lactoferrin, lysozyme, and gelatin are three common basic proteins known for their ability to interact with acidic proteins (lactoglobulin, ovalbumin, casein, etc.) and form various supramolecular structures. Their basic nature makes them highly promising for interaction with other acidic proteins to form heteroprotein complex coacervation (HPCC) with a wide range of applications. This review extensively examines the structure, properties, and preparation methods of these basic proteins and delves into the internal and external factors influencing the formation of HPCC, including pH, ionic strength, mixing ratio, total protein concentration, temperature, and inherent protein properties. The applications of different HPCCs based on these three basic proteins are discussed, including the encapsulation of bioactive molecules, emulsion stabilization, protein separation and extraction, nanogel formation, and the development of formulas for infants. Furthermore, the challenges and issues that are encountered in the formation of heteroprotein complexes are addressed and summarized, shedding light on the complexities and considerations involved in utilizing HPCC technology in practical applications. By harnessing the basic proteins to interact with other proteins and to form complex coacervates, new opportunities arise for the development of functional food products with enhanced nutritional profiles and functional attributes.

## 1. Introduction

Thanks to the functional quality and high nutritional value, protein is a crucial component in many foods and should be incorporated into the human diet [1]. As a major component in human tissues and organs, proteins are essential for maintaining tissue and organ growth, as well as promoting metabolism. Animal and plant proteins, which offer a balanced array of essential amino acids and superior nutritional benefits, serve not only as primary sources of protein but are also widely utilized in a variety of foods [2,3]. Amino acids give proteins amphiphilic and polyamphictic properties, which in turn enable them to interact with molecules with different properties. While globular proteins may exhibit limited overall flexibility, many of them contain unstructured and flexible sequences or domains that are essential for their proper folding and function. Certain proteins like RNases and cystatins utilize flexible hinge loop segments to form supramolecular adducts through mechanisms such as “3D domain swapping” [4,5]. Therefore, the coacervation of heteroprotein complexes involving globular proteins represents a specific instance of polymer complex coacervation.

**Figure 1:**
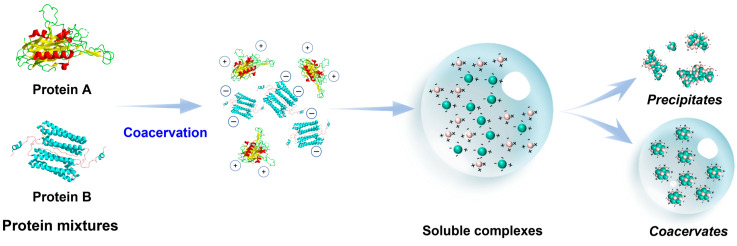
Schematic diagram of preparing heteroprotein complex coacervations based on acidic protein and basic protein with opposite charges.

Due to the limitations of specific properties and applications associated with individual proteins, researchers have recently focused on exploring the formation of heteroprotein complex coacervation (HPCC) using two proteins with opposite charges in the same solvent [5]. This phenomenon occurs due to the entropy increase resulting from the release of protein-soluble factors into a concentrated colloidal coacervate phase. This phase is stabilized by electrostatic interactions and exists alongside a dilute, protein-poor bulk phase. In HPCC, electrostatic attraction and desolvation lead to the formation of a two-phase system, typically characterized by phase separation between the proteins (Figure 1). By adjusting the pH of the system to the isoelectric point of the two proteins, one protein becomes positively charged while the other becomes negatively charged, enabling the induction of complex coacervation. When two biopolymers with opposite charges are mixed, the resulting complex usually consists of both soluble and insoluble components. The physical state of the coacervated phase varies between coacervated and precipitated forms and can be influenced by factors such as charge density and interactions between the biopolymers [6]. The protein–polysaccharide system is a typical example of a complex coacervation system [7], but there are few studies on the HPCC involving two proteins in this system. Different from traditional protein–polysaccharide systems, HPCC has unique performance attributes such as gelling and emulsification, which give it the ability to deliver active molecules, prepare emulsions and nanoparticles, and other aspects in the food field [8,9].

This review introduces three fundamental proteins commonly found in heteroprotein complex coacervation (HPCC) in food and medical fields: lactoferrin, lysozyme, and gelatin. The structure, properties, and extraction methods of these basic proteins are introduced in detail. These proteins possess unique properties that enable them to form heterologous protein complex coacervates through electrostatic interactions with acidic proteins under specific conditions. The internal and external factors influencing the formation of HPCC are discussed and the applications of different HPCCs based on these three basic proteins are reviewed. In addition, the current challenges, solutions, and the direction of future research are discussed. The primary focus is to explore the potential of proteins as delivery systems and draw inspiration from their application in various fields. By shedding light on the intricacies of these proteins and their potential in forming complex coacervates, this review aims to pave the way for innovative applications and further research in heteroprotein systems.

## 2. The Structure, Properties, and Extraction of Basic Proteins

Proteins are categorized into acidic and basic proteins according to their isoelectric point (pI). Specifically, proteins with a pI greater than 7 are considered basic, while those with a pI less than 7 are classified as acidic [10]. Current research primarily focuses on selecting an acidic protein and a basic protein to achieve stable complex coacervation across a broad pH range. This approach facilitates the investigation of the mechanisms underlying complex coacervation and its associated properties, enabling relevant application studies. Below are three commonly used basic proteins.

### 2.1. Lactoferrin

#### 2.1.1. Structural Characteristics of Lactoferrin

**Figure 2:**
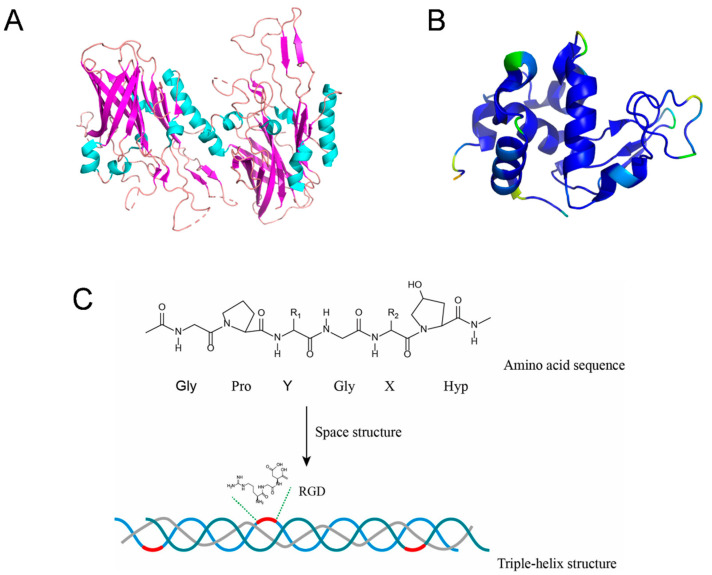
The 3D structures of (**A**) lactoferrin (PDB: 4U9C) and (**B**) lysozyme (PDB: 1TEW). (**C**) Constitution and structure of gelatin. Space structure refers to the protein formed by the winding and folding of the peptide chain [61]. RGD represents the arginine–glycine–aspartate sequence.

Lactoferrin (LF), a glycoprotein with a molecular weight of about 78 kDa derived from transferrin, is primarily extracted from the milk of mammals [11]. Importantly, different sources of LF exhibit distinct variants; for instance, human LF and bovine LF share only about 70% sequence identity [12]. As shown in Figure 2A, LF is folded into two globular lobes connected by an α-helical bridge and stabilized by four disulfide bonds [13]. LF has the function of regulating the concentration of free iron in biological fluids, which is mainly achieved by dissolving or sequestering Fe^3+^ [14]. Briefly, the secondary structure elements comprise 33–34% helices and 17–18% strands. Its overall structure is formed by connecting two symmetrical lobes (N-lobe and C-lobe) with an α-helix. Each lobe can be further divided into two sub-domains (N1 and N2; C1 and C2) [12,15]. Lacking Fe^3+^, the apo-LF easily undergoes protease action, while the holo-LF is more stable and resistant than the former to proteolytic attacks [16]. It is well known that the presence of free metal ions in the body can lead to the production of harmful free radicals and various other biochemical processes. Apo-LF can bind other metal ions, such as Co^3+^, Cu^2+^, Cr^3+^, Mn^3+^, Al^3+^, etc. [17]. Therefore, binding of these metals to apo-LF may potentially protect cells from oxidative-stress-induced damage.

#### 2.1.2. Properties of Lactoferrin

LF possesses various functional characteristics that stem from its molecular structure. Notably, the featured function of LF is its ability to bind iron. Moreover, LF can enhance intestinal iron absorption more efficiently than iron salts (Table 1) [12]. Studies have demonstrated that LF boosts nanoparticle toxicity, exhibits remarkable efficacy against cancer cells [18], and, when administered orally, impedes tumor progression and prolongs survival in mice [19], suggesting its potential as a preventive measure in cancer treatment. The use of liposomal LF through oral or intranasal routes has shown benefits in mild to moderate COVID-19 cases, indicating its practical utility in mitigating SARS-CoV-2 infection and highlighting its antiviral properties [20]. LF also exhibits free-radical-scavenging capabilities, can chelate copper (II) and ferrous (II) ions [21], and shows promise in protecting against kidney dysfunction induced by particulate matter [22]. Many studies support the notion that LF dietary supplements can enhance immune function due to their biological activities [23]. Furthermore, LF promotes osteoblast activity and inhibits bone resorption by influencing osteoclasts [24].

#### 2.1.3. Preparation of Lactoferrin

Initially, milk is defatted by centrifugation (3000× *g*, at 4 °C for 15 min). The pH is adjusted to 4–5 to separate milk protein and whey is collected by centrifugation. Finally, LF is recovered from whey by cation-exchange chromatography [25]. At present, cation-exchange chromatography is a common method for extracting LF, but this technology is limited by its high cost and relatively low yield [26]. Many previous technologies, such as gel filtration chromatography, immobilized monoclonal antibodies, and pressure-driven membrane processes also have disadvantages such as high cost, environmental hazards, and poor selectivity; thus, an innovative separation technology that can overcome the limitations is urgently needed [27]. In 2018, a novel competitive method, microbatch ion-exchange resin extraction, was introduced for quantifying LF in both raw and heat-treated milk. The first step involves quantitative extraction of lactoferrin using microbatches of resin, which prevents loss or dilution of the LF since the milk only needs to be skimmed. In the second step, LF recovered from the desorption supernatant using 2 M NaCl was completely separated from other cationic substances and was quantified at a wavelength of 220 nm using reverse-phase high-performance liquid chromatography. This method offers simplicity in operation, reduces dilution and loss of LF, and enhances both accuracy and sensitivity [28]. Furthermore, a new technique leveraging the iron-binding properties of LF is suggested for the selective extraction of the low-abundance glycoprotein LF from whey. This method involves optimizing the substitution between Tb^3+^ and Fe^3+^ to improve the adsorption and desorption processes. By utilizing the Fe vacancy domain of apo-LF, this approach can efficiently extract LF from complex substrates [29]. In summary, novel separation technologies can offer promising pathways to minimize the environmental impact of LF extraction. By reducing the number of reagents, simplifying processes, and maintaining high yields, these methods not only enhance the sustainability of LF production but also promote more environmentally friendly practices in the dairy-processing industry.

### 2.2. Lysozyme

#### 2.2.1. Structural Characteristics of Lysozyme

Lysozyme (LYS), an antibacterial enzyme, was discovered by Scottish bacteriologist Sir Alexander Fleming in 1921 and is widespread across various kingdoms, including animals, plants, and microorganisms [30]. This relatively small enzyme protein, existing primarily in monomeric form, is ubiquitous in nature, present in all living organisms, and exhibits bactericidal properties in all environments. Among them, there are three main types of LYS found in the animal kingdom: “type c” found in egg proteins; “type g” found in goose egg proteins; and “type i” found in invertebrates (Table 1) [31,32]. Current research primarily focuses on “type c” LYS, comprising 129 amino acid residues, with a molecular weight of 14.3 kDa and an isoelectric point (pI) of approximately 10–11 [33]. The presence of four disulfide bonds within the c-type LYS molecule enhances its thermal stability [34]. Previous studies have revealed that LYS molecules consist of two domains linked by elongated alpha-helices, with an interaction between Lys 97 and Phe 38 rendering the monomeric form hydrophobic within the molecule but hydrophilic on the surface [35,36]. In contrast, g-type LYS lacks n-terminal signaling peptides and cysteine residues crucial for disulfide bond formation, leading to differing intracellular functions in fish enzymes compared to their avian counterparts [37,38]. The discovery of type i LYS was initially reported in the invertebrate red sea star [39], exhibiting a structure akin to c-type LYS but with slight differences in the amino acid sequence and the presence of N-terminal and C-terminal regions. Notably, the N-terminal amino acid sequence resembles that of HEWL (Figure 2B). While various LYS variants exist within the same species, they display distinct enzymatic and structural characteristics, resulting in alterations in catalytic activity [40].

#### 2.2.2. Properties of Lysozyme

Egg white LYS, renowned for its exceptional stability, maintains its integrity even under extreme temperatures and in acidic environments. Demonstrating distinctive anti-inflammatory properties, this enzyme bestows advantageous effects on the immune system. Upon treatment with LYS, mouse macrophage-like cell line RAW264.7 and mouse peritoneal macrophages exhibited a notable reduction in tumor necrosis factor-α and leukocyte interleukin-6 production. These results suggest that LYS curbs the inflammatory response by inhibiting c-jun N-terminal kinase (JNK) phosphorylation within cells [41]. Moreover, LYS showcases substantial antioxidant activity, whether through enzymatic hydrolysis or in conjunction with specific active compounds (Table 1). Alkaline protease hydrolyzes LYS to yield peptides with enhanced antioxidant properties through a water-soluble free-radical-scavenging mechanism [42]. Peptic hydrolysis generates positively charged peptides with antioxidant capabilities, effectively reducing lipid peroxide oxidation by up to 63.2% [43]. By targeting the β-1,4-glucoside bond between N-acetylglucosamine and N-acetylmuramic acid, LYS hydrolyzes bacterial cell walls, leading to the degradation of the peptidoglycan structure and ultimately compromising the integrity of the bacterial cell [44]. Consequently, LYS exhibits antibacterial properties, as the negatively charged bacterial cell membranes interact with the positively charged LYS through electrostatic forces, driving their binding [45]. Moreover, the LYS system can be utilized to study the effects of various nanoparticles on the fibrillation spectrum, as it is associated with hereditary systemic amyloidosis. Insights into the mechanisms of lysozyme fibrillation can be extrapolated to other amyloid proteins in an effort to design therapeutic interventions for amyloid diseases [46].

#### 2.2.3. Preparation of Lysozyme

LYS exhibits excellent functional properties, making it a key focus in both pharmaceutical and food industries [46,47]. Consequently, the quest for an optimal LYS purification protocol remains a prominent area of investigation among scientists. Various techniques for LYS separation have been postulated, including ultrafiltration, precipitation, dialysis, and ion-exchange chromatography [48,49,50,51]. Nonetheless, the current methodologies exert significant strain on the environment and human well-being due to the elevated reagent toxicity, substantial costs, and intricate operational procedures. Given these concerns, there is a pressing need for more environmentally friendly and efficient methods for refining LYS. Recently, a sustainable liquid–liquid extraction method using a two-phase aqueous system (ABS) based on low eutectic solvents (DES) has emerged as a promising approach. Remarkably, this extraction method retains LYS activity at 91.73% of its initial level, demonstrating commendable biocompatibility [52]. In addition to the extensively explored polymer-based ABS, ionic liquids (ILs) represent salts comprising sizable organic cations and organic or inorganic anions. The principal advantage of IL as a phase-forming component of ABS lies in the capacity to tailor the polarity of the coexisting phase through manipulation of its ionic chemical structure, thus facilitating the design of enhanced ABS for specific applications. LYS extraction via a novel ABS incorporating alkyl ammonium ionic liquid and potassium phosphate solutions of varying pH values demonstrated the recovery of up to 99% of LYS from the IL-rich phase sans any structural alterations [53]. Furthermore, a straightforward approach entailed the extraction of LYS utilizing an ethylene-vinyl alcohol copolymer (EVOH) nanofiber membrane as the foundational material, with the subsequent preparation of a surface molecular imprinted matrix (MIP) predicated on the nanofiber membrane. The obtained LYS showcased substantial bioactivity [54]. The fabrication via chemical modification of carboxylated functional acrylonitron-crosslinked porous β-cyclodextrin polymers (P-CDPs) emerged as a method to produce a highly adsorbent medium for the selective extraction of LYS from egg white, successfully elevating the LYS adsorption from 615 mg g^−1^ of P-CDPs to 1520 mg g^−1^ of P-CDP-COO^−^ [55]. Overall, the transition towards these modern and sustainable extraction techniques holds the potential to significantly reduce waste generation and energy consumption, while maintaining high efficiency and biocompatibility essential for pharmaceutical and food industry applications. By using eco-friendly materials and innovative methods, the ecological footprint of LYS purification can be significantly reduced, promoting sustainability in the food industry.

### 2.3. Gelatin

#### 2.3.1. Structural Characteristics of Gelatin

Gelatin is a protein resulting from the partial hydrolysis of collagen. It exhibits a similarity in amino acid composition and homology to its collagen precursor. The hydrolysis process of collagen into gelatin involves the deamination of glutamine and asparagine. Gelatin has a relative molecular weight that ranges from 15 to 250 kDa, which influences its properties and applications. Lower-molecular-weight gelatin (around 15–50 kDa) tends to exhibit higher solubility and faster gelation, making it suitable for applications requiring quick setting, such as desserts and confections. In contrast, higher-molecular-weight gelatin (above 100 kDa) provides greater gel strength and improved textural properties, making it ideal for products like gummies and marshmallows (Table 1). Moreover, gelatin predominantly originates from animal sources such as skin, bone, and tendon, and it can also be extracted from discarded leather remnants [56]. Notably, two distinct classifications of gelatin, namely type A gelatin and type B gelatin, are delineated by their respective pretreatment methods, encompassing acid or base conditions. Type A gelatin is generally preferred in acidic foods and clear products, while type B gelatin is suited for neutral to alkaline conditions and provides stability in dairy and cosmetic products. Understanding these differences allows manufacturers to choose the appropriate type of gelatin for specific applications to optimize both performance and product quality. Enzymatic pretreatment constitutes a pivotal phase in gelatin production, targeting specific labile peptide bonds to ensure optimal product quality [57]. The variances in pretreatment methodologies and extraction techniques yield peptides of diverse conformations and sizes. Collagen undergoes structural alteration and denaturation during the gelatinization process, culminating in the formation of a triple-helix configuration [58]. The gelatinous triple helix, formed by intertwined polypeptide chains, solidifies into a gelatin gel as intermolecular interactions create a conjugation zone when the chains cool [59]. Subsequently, the gelatinoid triple-helix structure undergoes partial dissociation and rearrangement, with hydrogen bonding, electrostatic forces, and hydrophobic interactions identified as pivotal factors in stabilizing these triple helices [60]. Additionally, gelatin, which serves as an analogue of the extracellular matrix, contains a distinct arginine–glycine–aspartate (RGD) sequence (Figure 2C) that fosters an optimal biological environment for bone cell adhesion, proliferation, and biomineralization [61].

#### 2.3.2. Properties of Gelatin

The gel strength, viscosity, gel point, and melting point of gelatin are crucial physical properties in food applications (Table 1). Among these, gel strength is the most significant index for evaluating gelatin quality. Gelatin strength values below 150 g are considered low, while those ranging from 150 to 220 g are classified as medium, and values between 220 and 300 g are deemed high. Variations in gelatin properties are notable across different sources. For instance, fish gelatin exhibits a gel strength range of 70–270 g and a melting temperature range of 11–28 °C. In contrast, mammalian gelatin typically features a gel strength of 200–400 g, a gel point of 20–25 °C, and a sol point of 28–31 °C [62]. Mammalian gelatin features a more compact and uniform structure, resulting in superior emulsification and foaming properties compared to fish gelatin. Its higher hydroxyproline and proline content leads to elevated gel melting temperatures. Gelatin exhibits a porous and loose network structure, with abundant hydrophilic groups and strong water-holding capacity. It can be readily dissolved in warm water and showcases excellent solubility, making it a popular food additive in candies, jellies, and yogurt [56]. Temperature significantly influences the rheological properties and viscosity of collagen-based foods [63]. Gelatin transitions from a liquid state at high temperatures to a gelatinous solid at lower temperatures, highlighting its thermal reversible nature. In addition, its thermal stability depends on the amino acid content, which stabilizes the gelatin’s tertiary structure through constraint from proline and hydroxyproline and hydrogen bonding by the hydroxyl groups of hydroxyproline [64].

**Table 1:** A summary of the sources, molecular weight, and properties of three basic proteins (lactoferrin, lysozyme, and gelatin).

Basic Proteins	Source	Molecular Weight	Properties	References
Lactoferrin	Milk of mammals	78 kDa	Binding iron; anticancer activity; antitumor activity; antiviral properties; free-radical-scavenging capabilities; immune function	[11,18,19,20,21]
Lysozyme	Animals; plants; microorganisms	14.3 kDa	Antioxidant activity; anti-inflammatory properties; antibacterial properties; fibrillation	[30,33,41,42,45,46]
Gelatin	Animal sources such as skin, bone, and tendon	15–250 kDa	Emulsification; foaming properties; water-holding capacity; solubility; water absorption; rheological properties; thermal stability	[56,63,64]

#### 2.3.3. Preparation of Gelatin

Gelatin is typically derived from the skin tissues or bones of animals like pigs, cattle, fish, and poultry [65]. The industrial processes for its extraction encompass several techniques, including solid–liquid extraction, percolation, acidification, and gelatin impregnation [66]. These methods are employed to eliminate impurities and minerals through demineralization, as well as non-collagenous materials. Subsequently, the hydrolysis method is the key chemical process utilized to convert collagen into gelatin [67]. This process involves breaking down the collagen’s triple-helical structure, leading to the formation of smaller peptide chains. In biological processes, enzymatic extraction methods are considered more promising for hydrolysis processes [66]. The enzyme extraction method offers the benefits of a brief processing period and reduced waste generation, yet it is accompanied by the drawback of elevated extraction expenses. In the enzymatic gelatin extraction process, various proteolytic enzymes facilitate the conversion of collagen into gelatin. For example, an optimal extraction includes the use of pepsin (547 U/g), a temperature of 46.98 °C, a pH level of 4, and a duration of 1.27 h [68]. Furthermore, the quality and yield of gelatin are influenced not only by the source of the collagen but also by the extraction method and conditions employed during the process. This occurs because the bonds within the polypeptide chains of collagen are disrupted during hydrolysis, leading to the breakdown of its fibrous structure and the production of gelatin [69]. The initial acidic extraction method involves utilizing an acid solution to catalyze the hydrolysis of collagen material, resulting in the production of type A gelatin. This technique is frequently employed for extracting collagen from pig skin, involving treatment with phosphoric acid or acetic acid solution for a duration of 10–45 h [70]. This extraction approach amplifies the collagen’s swelling properties, leading to improved hydrolysis and a heightened yield percentage [71]. However, acid treatment generates many environmental concerns, such as production of harmful substances, and loss of amino acid interactions with the consequent denaturation. Ultrasonic treatment disrupts cells through acoustic cavitation, enhancing the mass transfer of cell contents and consequently yielding a higher extraction of gelatin. Research indicates that cowhide gelatin hydrolysate treated with 300 W ultrasonic power exhibits robust antioxidant properties and resilience [72], but ultrasonic treatment is usually accompanied by high energy consumptions. Additionally, the extraction time can be slashed by over 50% through the application of a high-pressure extraction method [73]. This non-thermal technique leverages high pressure to disrupt non-covalent protein interactions, leading to protein denaturation and thereby facilitating the extraction of gelatin proteins [74]. The gelatin extracted from chicken skin using ohmic heating (OH) exhibits superior water- and oil-binding capabilities, along with enhanced emulsification and foaming properties. This method boasts a shorter extraction duration, increased yield, improved functionality, and heightened thermal stability compared to conventional extraction techniques [75]. Therefore, novel extraction methods, such as enzymatic, high-pressure, and ohmic heating, provide environmentally friendly alternatives to traditional gelatin extraction by reducing chemical use and waste. By adopting these techniques, the gelatin industry can lower energy consumption while enhancing product quality and yield.

## 3. Factors Affecting the Formation of Heteroprotein Complexes

**Figure 3:**
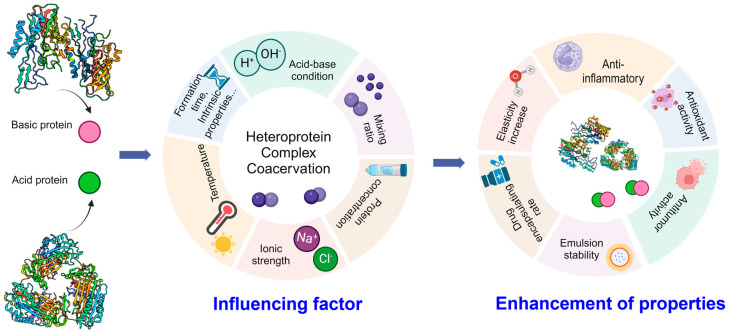
Factors (including pH, mixing ratio, protein concentration, ionic strength, temperature, etc.) influencing the formation of heteroprotein complex coacervates, along with the enhanced properties of these complexes compared to individual proteins.

The influencing factors in the formation of HPCC are shown in Figure 3. In the realm of food processing, HPCC plays a vital role due to its unique ability in enhancing the texture, stability, and nutrient delivery of food products. The food substrate is notably susceptible to the influence of environmental and various other factors. The primary catalyst for the liquid–liquid phase separation of HPCC is the electrostatic interplay between the two proteins, which is dynamically impacted by an array of variables such as pH, mixing ratios, salt properties (concentration, valence, and type), temperature, incubation duration, and the structural attributes of proteins. Understanding these variables is crucial as they not only influence the formation and stability of HPCC but also affect the overall quality and functionality of the food products. The ability of HPCC to form stable emulsions and foams makes it essential in improving the mouthfeel and sensory attributes of foods. Additionally, HPCC can encapsulate bioactive compounds, thereby enhancing nutrient delivery and promoting health benefits, which is important in the development of functional foods.

### 3.1. Acid–Base Condition

**Figure 4:**
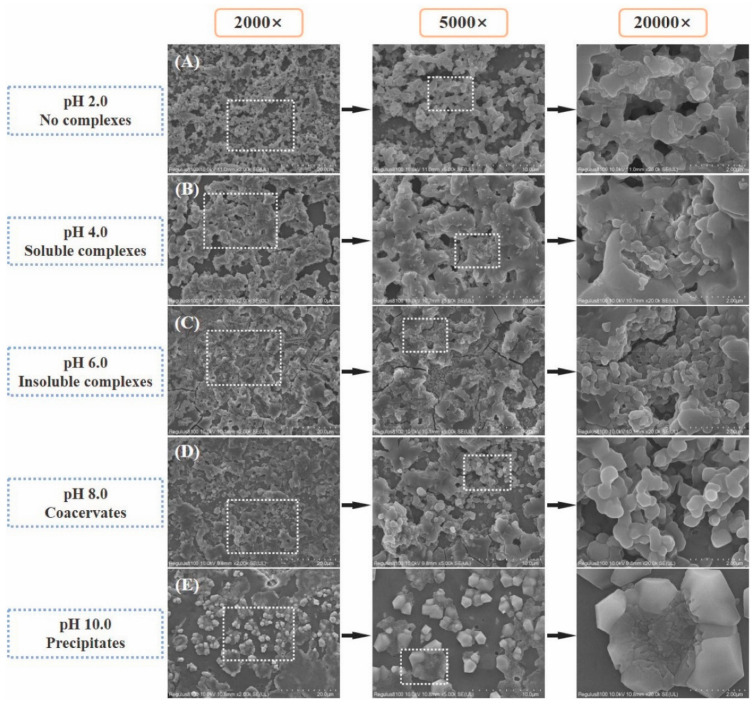
SEM micrography of the OVA-LYS complexes at five pH values (pH 2.0/4.0/6.0/8.0/10.0). The OVA-LYS complex structures were critically dependent on environmental pH [80]. (**A**–**E**) SEM micrography of sample at pH 2.0, 4.0, 6.0, 8.0, and 10.0, respectively.

The pI of the protein and the pH of the solvent are crucial factors in determining the protein’s surface charge. By manipulating the pH, the dissociation level of the protein’s side chain groups can be controlled, leading to alterations in the protein’s charge. pH adjustment can effectively control the electrostatic interaction between heteroproteins. At acidic pH levels (below the pI of the protein), there is typically an increase in electrostatic repulsion between acidic and basic proteins, which reduces the likelihood of complex formation. Conversely, under alkaline pH conditions (above the isoelectric point), basic proteins may lose their positive charge, resulting in a net neutral or even slightly negative charge. This allows the amino and carboxyl groups of the protein’s side chain to undergo protonation and deprotonation processes [76]. The interaction between β-lactoglobulin (β-LG) and LF was observed to induce liquid–liquid separation and increase association or aggregation at pH 5.7–6.2. This cannot be attributed to LF with reduced turbidity in this range but must be attributed to the formation of one or more balanced β-LG-LF complexes. When the pH was between 7.5 and 10.0, β-LG (pI~5.2) inhibited the aggregation of LF (pI~8.7), whereas at pH 4 to 5, LF inhibited the aggregation of β-LG; in these conditions, the turbidity of the mixture was lower than the sum of the individual components [76]. In the early stages, biopolymer molecules often create soluble complexes within themselves. Subsequent electrostatic interactions result in a dense and viscous liquid phase known as coacervate, emerging from a homogeneous macromolecular solution with limited solubility due to thermodynamic incompatibility. In an LF–pea protein isolate (PPI) mixture, the hydrodynamic radius remained below 75 nm at all pH levels, except for pH 5 to 6. At a pH of approximately 5.4, a larger aggregate formed, reaching a maximum size of about 80 nm [77]. This indicates that the interaction between LF and PPI does result in the formation of a new “liquid” (coacervate) phase. Furthermore, another study involved titrating 5 mg/mL of LF into 5 mg/mL of β-LG and the pH was a function of the [LF]/([β-LG] + [LF]) ratio [78]. Initially, the pH increased (pH~6.73), then decreased (pH~6.48), before returning to its original level. This behavior is due to charge regularization. The ongoing addition of LF to the system resulted in excessive binding, causing the excess β-LG bound with LF to release some protons, leading to a slight decrease in pH value [78]. The assembly of the two proteins strictly adheres to the principle of electrostatic compensation, a characteristic observed in all publicly available LYS-based heterozygous protein complexes. Zheng et al. explored the impact of mixing ratio on the turbidity of a soy protein isolate (SPI)/LYS complex within the pH range of 6.5–8.0 [79]. With the pH increasing from 6.5 to 8.0, the stoichiometry of the complex (SPI/LYS) underwent a significant reduction due to the charge variations of SPI and LYS. In other words, at higher pH levels, there was a greater presence of LYS in the optimal complex. As illustrated in Figure 4, the configuration of ovalbumin (OVA)–LYS complexes is greatly influenced by the pH of the environment [80]. As the pH level rises, soluble complexes progressively transform into insoluble complexes or coacervates. The rising pH alters the charge properties of proteins and diminishes electrostatic repulsion. In addition, this pH alteration also enhances hydrophobic interactions and modifies the solubility of protein complexes, ultimately leading to the formation of insoluble complexes or coacervates [80].

### 3.2. Mixing Ratio

The mixing ratio of different proteins is crucial for regulating the protonation and deprotonation of amino and carboxyl groups in protein side chains, and it can in turn affect the charge balance among proteins and formation of heteroprotein complexes. For instance, at pH 5.0 and 5% (*w*/*v*) protein, the LF:osteopontin (OPN) mixing ratio (based on protein mass) ranges from 2.5 to 7.0 [81]. This influence extends to phase separation, where the mixing ratio of protein components may differ from the stoichiometric ratio in the coacervated phase. This discrepancy often arises from the overrepresentation of one protein within the complex [81]. Despite variation in mixing ratios, the protein stoichiometry in the condensed layer remains relatively consistent, typically showing LF in excess compared to OPN. Further differentiating phase behaviors, liquid–liquid phase separation is characterized by the formation of a viscous coacervate phase, often described as having a “honey-like” consistency. This phenomenon contrasts with liquid–solid phase separation, where significant desolvation occurs during the precipitation process, leading to partial desolvation of the protein complex [82]. Importantly, the coacervated phase formed is reversible; changes in the pH or ionic conditions of the solvent can lead to the dissolution of the separated coacervated layer (Table 2) [81,83].

In a study examining the interaction between LYS and OVA, the highest turbidity was observed at LYS:OVA ratios of 1:1 and 2:1, which corresponded to an increased amount of coacervate complexes [84]. This phenomenon is attributed to the neutralization of negatively charged groups in OVA molecules by the positively charged groups in LYS molecules at a 1:1 ratio. This charge neutralization is crucial, as the protein proportions significantly influence turbidity by maintaining charge balance and determining the strength of complex formation [84]. In a separate analysis involving BSA-GB, β-LG-GB, and BSA-GB-β-LG pairs, it was noted that absorbance levels remained constant when the ratios reached a certain threshold [85]. This constancy indicates that no structural changes occur at these ratios, which are deemed the maximum binding conditions for all pairings. These findings highlight the interplay between protein ratios and structural stability in complex formations.

**Table 2:** Properties, types, experimental index, and potential applications of heteroprotein complexes formed based on lactoferrin.

Basic Protein	Interacting Protein	pI	Procedure to Prepare Heteroprotein Complexes	Application	References
Lactoferrin (pI~8.7)	β-LG	5.2	Mass ratio β-LG:LF = 1:1, salt concentrations < 20 mM, pH = 5.7–6.2	-	[76]
	PPI	-	pH 5.0–5.8	-	[77]
	β-LG	4.8	Stoichiometry LF: β-LG = 1:3, pH = 5–7.3	-	[78]
	OPN	-	pH (~4–6), ionic strength (≤30 mM added NaCl), protein stoichiometry (LF:OPN mass mixing ratios of ~2–8), total protein concentration (≤~8% *w*/*v*)	-	[81]
	β-LG	5.2	β-LG/LF molar ratio of 10, pH = 5.5	As biocarrier for vitamin B9	[86]
	BCN	4.6–4.8	Salt concentrations < 0.4 mM	-	[87]
	β-LG	5.2	Protein molar ratio of 2:1 LF/β-LG, pH = 6.5, temperatures of 62.5 °C	-	[88]
	TG	-	100 U/g of TG concentration, 50 °C, 2 h of crosslinking time	Pickering emulsions with improved curcumin bioaccessibility	[89]
	GMP	4–5	Molar ratio LF:GMP = 1:7, pH = 5.0, heated at 80 °C	Nanohydrogels	[90]
	β-LG	-	Protein concentration of 26% *w*/*w*, with a 2:1 ratio of BLG:LF, a final B9 loading of 0.04% *w*/*w*, pH = 5.5	As biocarrier for vitamin B9	[91]
	API	-	Encapsulation was conducted at pH 6.5 and a ratio of 1:3 (API: LF, *w*/*w*)	For VD3 encapsulation and fortification of bakery products	[92]
	β-LG	4.5–5.5	Mass ratio β-LG: LF = 1:1, pH = 7	Oil-in-water emulsions	[93]
	WPI	4.9	Mass ratio WPI:LF = 1:1, 1% (*w*/*w*) LF and WPI, pH = 6	Stability of nanoemulsions	[94]
	β-LG	5.2	pH = 5.4–6.0	A high yield of LF recovered	[95]
	BSA	4.9	BSA permeation flux of 30.31 g m^−2^ h^−1^, LF permeation flux of 1.07 g m^−2^ h^−1^	Identify the most effective conditions for protein separation	[96]
	OPN	-	Molar ratios of LF:OPN = 3:1, 5:1, or 8:1	Infant formula	[97]
	OPN	-	Apo-LF or holo-LF with apo-OPN or holo-OPN at a molar ratio of 3:1	Infant formula	[98]

“-” represents missing information or work not reported.

### 3.3. Protein Concentration

**Figure 5:**
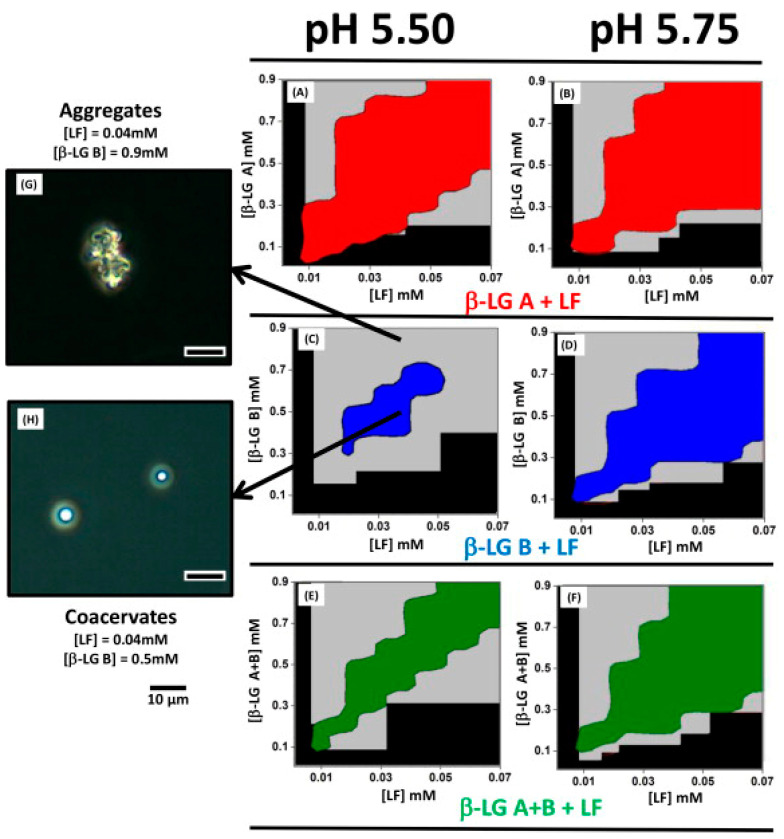
Phase boundaries of coassembly of LF with β-LG isoforms at pH 5.50 and pH 5.75. (**A**,**B**) LF/β-LG A; (**C**,**D**) LF/β-LG B; (**E**,**F**) LF/β-LG AB. Black zones: domains without detectable supramolecular structures; Gray zones: aggregation domains. Red, Blue, and Green zones: coacervation domains. Optical microscopy of aggregates (**G**) formed by mixing, for example, 40 mM LF and 900 mM β-LG B at pH 5.50 versus coacervates (**H**) formed by mixing, for example, LF 40 mM and β-LG B 500 mM at pH 5.50 [95].

The total protein concentration is also a factor that influences the formation of complex coacervation. It can alter the interaction potential energy and assembly dynamics among proteins. Yan et al. reported the formation of LF+β-LG coacervate and precipitate at a protein concentration of 60 g/L, with coacervation competing with aggregation at protein concentrations ranging from 10 to 40 g/L [76]. Specifically, coacervates can be formed under specific conditions of protein concentration. The concentrations needed for coacervation are disproportionate; a significant excess of β-LG, the negatively charged protein with a lower molecular weight, is necessary. A coassembly of the aggregate type was observed at a protein concentration (LF+β-LG) of 1.1 mM, whereas a coassembly of the coacervate type was formed at a protein concentration of 0.55 mM. In Figure 5, the conserved domains of the β-LG A, β-LG B, and LF mixtures overlap at both pH values [95]. The coacervated domains of β-LG A and LF were notably larger than those of the other two mixtures, completely enveloping them. The combination of β-LG B and LF exhibited the smallest coacervation domain at pH 5.50. At pH 5.50, LF/β-LG coacervation only occurred within a protein concentration range of approximately 0.02–0.05 mM. Beyond this concentration range, LF inhibition of aggregation takes place, leading to a transition from coacervation to aggregation. In polyelectrolyte systems, the disappearance of coacervates above the critical polymer concentration is well-documented and predicted by continuously modified coagulation theories [95]. In addition, β-casein and LYS nanoparticles at a concentration of 0.1 mg/mL were stable in aqueous solutions at both pH 5.0 and pH 10.0 [99]. After one month of storage, there was no significant change in the size distribution of the nanoparticles. Furthermore, nanoparticles can be prepared at higher protein concentrations, such as 10 mg/mL.

### 3.4. Ionic Strength

The ionic strength plays a significant role in the behavior of complex proteins, primarily due to the electrostatic interactions between groups carrying opposite charges. A notable synergistic enhancement of the protein complex is evident in the presence of ions, which have the capability to modify the surface charge density of the molecule, as observed in a study by Wong et al. [100]. When salts, such as NaCl, are introduced into the solution, Na^+^ ions compete with positively charged groups of proteins and associate with the negatively charged sites, while Cl^−^ ions compete with the negatively charged sites and bind to the positively charged regions. Therefore, the ions can change electrostatic interactions between two oppositely charged proteins at certain pH and in turn influence the formation of heteroprotein complexes [100].

In the complex coacervates of LF and β-LG, the addition of NaCl resulted in a weakening of the complexation, with the complete disappearance of the complexation observed at a salt concentration of 100 mM. Yan et al. reported that the aggregation-induced free β-LG consumption effectively competed with higher-order β-LG-LF complexation during the transition from high-salt to low-salt conditions [76]. β-LG aggregation prevailed at elevated salt concentrations not due to its intrinsic speed but rather because complex formation was significantly diminished. Additionally, 8-anilinonaphthalene-1-sulfonic acid (ANS) did not interact with α-LG, but it exhibited binding to LF through two distinct sets of binding sites, prompting self-aggregation of LF. The hydrodynamic increase in the ANS/LF complex was moderately influenced by the rise in ionic strength upon the addition of 50 mM NaCl. In an isothermal titration calorimetry (ITC) experiment, the introduction of 50 mM NaCl notably decreased the enthalpy of the first injection and the exothermic signal associated with the second binding zone. Higher concentrations of NaCl further diminished the signal strength in both regions [101]. The coacervation of LF and osteopontin (OPN) (r = 4:1 pH 5.0, 5% *w*/*v* protein) was impeded by NaCl concentrations exceeding 30 mM. With each increment in ionic strength compared to the control sample (no salt), there was a reduction in the coacervation yield and the concentration of the condensed protein, leading to the formation of a noticeably weaker and less viscous coacervation phase [81].

The formation of β-CG/LYS complex coacervates occurs within the range of 5–80 mM NaCl at pH 6.0 and 7.0 and 40–80 mM NaCl at pH 8; furthermore, the presence of 100 mM NaCl almost entirely inhibits the recombination of β-CG/LYS [102]. In the presence of 20–40 mM NaCl, the turbidity of the β-CG/LYS coacervates formed across a broad pH and mass ratio spectrum. Following a rapid ascent to the peak value, the turbidity of these coacervates gradually diminished [103]. Research on the incorporation of curcumin into β-CG-LYS complexes revealed that the inclusion of 0–40 mM NaCl did not diminish the encapsulation efficiency of curcumin. The efficiency remained consistently high, ranging from 96.5% to 96.7% across pH levels within pH 6.0 and 8.0. Curcumin was predominantly found within the condensed droplets [104]. The surface hydrophobicity of SPI/LYS composite coacervates decreased with increasing NaCl concentration, with a critical concentration of 200 mM, higher than that of heteroprotein coacervates but lower than that of non-heteroprotein coacervates [105]. The inclusion of 200 mM NaCl entirely hindered the electrostatic assembly between SPI and LYS (1 mg/mL). NaCl diminished the electrostatic repulsion between colloids, leading to a substantial increase in the size of SPI/LYS complexes with the presence of 5 mM NaCl. Moreover, the average size of nearly all complexes escalated to micron dimensions in the presence of 20 mM NaCl [79]. β-casein-glucan-LYS nanoparticles were synthesized by attaching glucan to β-casein through a Maillard reaction [99]. While the complex micelles showed instability in the presence of NaCl, the nanoparticles remained stable in solutions with a pH range of 5.0–12.0 and a NaCl concentration of 0.15 M [99].

The inclusion of MgCl_2_ has been shown to significantly decrease the critical gel concentration of the protein, potentially attributed to the specific binding of the bivalent ion to amino acids on the protein surface. The lower G’ (storage modulus) observed in the NaCl-added sample compared to the MgCl_2_-added sample indicates incomplete cogelation of the OVA-LYS heteroprotein complex under these conditions. This phenomenon provided more evidence that divalent ions could enhance the molecular crosslink and decreased the critical gelation concentration of samples [106]. Therefore, this observation further supports the notion that bivalent ions enhance molecular crosslinking and reduce the critical gel concentration of the sample. At various NaCl concentrations with a ratio of r = 1, the presence of NaCl pairs exerted a detrimental impact on the development of the OVA-LYS coacervate complex. This effect was noticeable even at low concentrations (10 mM) and became more pronounced at higher concentrations (300 mM), inhibiting the interaction between the components [84]. Similarly, researchers found that NaCl negatively impacts the formation of BSA/LYS coacervates. Even at low concentrations of 10 mM and 50 mM, there is a reduction in turbidity intensity and a narrower range for complex formation [107]. In the process of preparing gelatin and gelatin microcapsules, there was a slight increase in coacervation yield as the ionic strength rose from 0 to 0.1 mM. However, a subsequent increase in ionic strength resulted in a rapid decline in coacervation yield, with complete inhibition of coacervation occurring at an ionic strength of 5.0 mM (Table 3) [108].

### 3.5. Temperature

The physical state of the complex formed after the binding phase separates can either be a liquid coacervate or a solid precipitate. This state is influenced by the kinetics of the composite coacervation process. Importantly, the properties of the coacervated phase are affected not only by the chemical characteristics of the charged materials but also by external factors such as temperature. Temperature appears to have a lesser impact on the coacervation of oppositely charged polymers and tends to be more closely related to the properties of individual proteins [115].

Research conducted by Anema and de Cruif on casein and LF revealed that the scattering intensity of the BCN/LF system decreased by a factor of two within the temperature range of 5–35 °C [87]. This finding suggests that the hydrophobic tail of BCN influences the complexation process, as BCN operates as a monomer at 5 °C but transitions into micelles by 35 °C. In the investigation involving LF and β-LG, researchers measured the scattering intensity of the sample’s maximum turbidity at pH 6.3 while varying the temperature [78]. They observed that the scattering did not exhibit a consistent pattern with temperature variations. Consequently, it was deduced that the interaction between the two proteins is primarily electrostatic and not significantly influenced by a low temperature. The ideal temperature for achieving a supramolecular structure in LF/β-LG is 62.5 °C [88]. At this temperature, a stable supramolecular protein structure is established. However, raising the temperature to 80 °C leads to a change in the secondary structure. As the reaction temperature rises, the particle size also increases. This phenomenon occurs because, within a specific temperature range, increasing temperature enhances transglutaminase (TG) activity and Brownian motion between LF molecules, thereby accelerating the reaction rate. After being maintained at 50 °C for 2 h, the particle’s size reached its maximum [89]. As the temperature increased, the aggregation of LF and glycomacropeptide (GMP) mixtures intensified, resulting in a reduction in both the hydrodynamic diameter and the polydispersity index (PdI) of the particles. Overall, these findings highlight the complex interplay between temperature and the physicochemical properties of protein interactions during coacervation [90].

An increase in reaction temperature usually leads to a corresponding increase in particle size. The interaction between α-lactalbumin (α-LA) and LYS gives rise to various supramolecular structures at different temperatures. At 5 °C, heterogeneous, amorphous aggregates are produced, while at 45 °C, spherical droplets form. Prolonged heating induces formation of larger aggregates through a coalescence process [109]. In the temperature range of 298 to 318 K, the enthalpy change rises as the temperature increases. Conversely, in the range of 318 to 328 K, the enthalpy changes decrease with rising temperature. At around 318 K, sodium caseinate (CAS) and LYS are exposed to more oppositely charged regions compared to other temperatures. The interactions between these two proteins are influenced by temperature fluctuation and primarily driven by entropy. The temperature can change the hydrophobic interactions and hydrogen bonds that significantly contribute to the interactions between CAS and LYS. Variations in temperature can modify protein structure and subsequently impact the interaction between these two proteins to form heteroprotein complexes [110]. 

In the LYS/gelatin A system, the creation of water-insoluble composite particles is entirely prevented at temperatures exceeding 35 °C. Across a broad temperature range (38–58 °C), no phase separation was detected in the LYS/gelatin A system [116]. In another study, gelatin A and B coacervation exhibit temperature-dependent characteristics, with the melting temperature of the coacervation layer being ≥42 °C. Gelatin gels are formed through intermolecular hydrogen bonding, whereas the coacervate layer originates from robust electrostatic interactions. This elucidates the elevated melting temperature linked with the coacervate layer [117]. The β-LG-gelatin B coacervated layer samples were heated slowly to a peak temperature of 45 °C and subsequently cooled gradually to 20 °C. Throughout this procedure, all the opaque coacervate layer samples transitioned into a cloudy sol, which, after a 24 h equilibrium period, transformed into a gel-like network (Table 4) [118].

### 3.6. Other Factors Influencing the Structure and Properties

Several potential factors, including the formation time, properties of the proteins engaged in heteroprotein coacervation, metal ligand state, denaturation state, posttranslational modification, charge density, and chain length of the polypeptide, can influence the structure and properties of the heteroprotein complex. The size of the LF/β-LG complex progressively grows over the initial 2 h, eventually resulting in phase separation and precipitation [86]. Upon encapsulation of B9 in the LF/β-LG complex, a supramolecular structure formed spontaneously within 1 min, with opacity and density increasing within 5 h. This coacervation process can be characterized as a spontaneous coassembly process. Furthermore, a similar phenomenon was noted in the LYS/apo α-LA assembly, where clusters of nanoparticles were observed just 30 s after mixing [111]. These clusters of supramolecular structures underwent continuous rearrangement over time, being influenced by thermodynamic effects. Furthermore, coacervated microspheres characteristic of the system appeared at the 4 min mark and achieved uniformity by the 6 min point, suggesting a dynamic equilibrium at that juncture [111]. In the interaction between LYS and OVA, it was observed that the addition of NaCl or acylation of LYS can inhibit its aggregation [112]. Lysine residues in LYS played a direct role in the polymerization process, while cysteine residues in OVA did not contribute to the aggregation of LYS. Moreover, the charged polypeptide polylysine displays a random coiled conformation at a suitable pH due to the rejection of the charged side chain. When the charge was neutralized at high pH, these peptides could adopt α-helices similar to the natural hydrophobic polypeptide polyalanine [120], and the α-helical structure of the polypeptide inhibited the formation of β-sheets, thereby preventing solid-state formation.

## 4. Applications of the Basic Protein-Based Heteroprotein Complex Coacervations

### 4.1. Encapsulation

Once HPCCs are formed, they can significantly enhance the properties and functionalities of the individual proteins involved in the complex (Figure 3). Many studies indicate that bioactive compounds, apart from fulfilling essential nutritional requirements, can potentially lower the risk of various diseases like cancer, inflammation, and cardiovascular issues [121]. Nonetheless, the absorption of oral bioactive compounds within the body is notably limited, as they can undergo degradation by enzymes in challenging environments such as the gastrointestinal tract, leading to hydrolysis that compromises their integrity [122]. The utilization of nano-embedded delivery systems can present encouraging possibilities by using protein-based nanocarriers to enhance their stability and bioavailability of the bioactive compounds [123]. The heteroprotein complex coacervation system shows excellent prospects in the effective encapsulation and targeted delivery of various types of nutritional supplements. Folic acid, also referred to as vitamin B9, plays a crucial role in numerous essential biochemical processes and is integral to the biosynthesis of various amino acids [124]. Regrettably, humans lack the ability to synthesize B9 internally, necessitating the inclusion of sufficient B9 sources in the daily diet [125]. LF-β-LG coacervates have demonstrated exceptional efficacy as carriers for B9 in biological systems, with optimal performance observed at an embedding ratio of 10 mg B9 per gram of protein. These B9-LF-β-LG coacervates exhibit spontaneous formation, exhibit relative stability, and are easily producible, albeit showing slight coalescence over time [86]. During UV irradiation and H_2_O_2_ oxidation, researchers discovered that β-LG-LF coacervation effectively shields B9 from chemical degradation. Additionally, the B9-loaded coacervates exhibited significant physical stability within the food substrate. When compared to unencapsulated B9, oral administration of B9 coacervated in healthy rats led to increased plasma levels of the vitamin. This enhancement primarily stemmed from the improved solubility of B9 throughout the gastrointestinal tract [91].

Vitamin D3 (VD3), a fat-soluble precursor being crucial for regulating phosphorus and calcium homeostasis, also exhibits immunomodulatory effects that help to protect the body from infections and certain inflammatory processes [126]. VD3 was encapsulated within a coacervated complex microcapsule formed by amaranth protein isolate (API) and LF [92]. These microcapsules were spherical, with a particle size of less than 600 nm, and achieved an encapsulation efficiency of up to 90%. In simulated gastrointestinal digestion conditions, approximately 70% of VD3 was released at the intestinal stage, with a bioaccessibility of around 46%. Microencapsulation demonstrated superior protection for VD3 compared to its unencapsulated form, safeguarding it from degradation due to UV radiation and room temperature storage for up to three times longer [92].

Curcumin, a hydrophobic, heat-sensitive, and photosensitive food pigment, has garnered significant attention for its antioxidant and anti-inflammatory properties, making it a prominent natural active compound. Complex coacervates and precipitates were created from β-conglycinin (β-CG) and LYS [104]. The β-CG-LYS complex exhibited coacervate behavior at pH 6 and precipitate behavior at pH 7.0 and 8.0. The encapsulation efficiency of curcumin reached 95%, with a drug loading ranging from 410 to 486 μg/mg. Both the coacervates and precipitates notably enhanced the stability of curcumin when subjected to light and heat exposure. During storage, curcumin within the coacervate displayed an exceptionally long half-life, whereas curcumin in the precipitate exhibited a shorter t_1/2_ value [104]. Fucoxanthin (FX) is a marine carotenoid with a unique structure that imparts various biological activities beneficial to human health, such as lipid-lowering, weight loss, antidiabetic, and anticancer effects [127]. The non-covalent interaction between FX and whey protein isolates (WPIs) leads to the spontaneous formation of nanocomplexes. Subsequently, the binding of whey protein isolate with FX and LYS resulted in the formation of coacervation complexes through electrostatic interactions. It was observed that the composition of miscellaneous proteins and pH levels influenced the coagulation process, leading to changes compared to free whey protein isolate. This heteroprotein system exhibited higher loading efficiency and offered enhanced protection for FX in conditions involving heating, storage, and simulated gastrointestinal environments [113].

*Lactobacillus reuteri*, a probiotic culture that is part of the human and animal gut microbiota, offers numerous benefits such as reducing the duration of diarrhea in children. It functions in regulating gut health, alleviating digestive discomfort, and improving symptoms of irritable bowel syndrome [128]. However, the challenge lies in obtaining probiotic cells. The potential for encapsulating probiotic *Lactobacillus reuteri* using heteroprotein coacervation complexes (type A gelatin/sodium caseinate, GE/CAS) was compared with protein/polysaccharide complex coagulation (type A gelatin/Arabian gum, GE/GA). Optimal pH and GE/CAS ratios for forming water-in-water emulsions were identified at pH 6.0 and GE/CAS = 2.0. Moreover, *Lactobacillus reuteri* maintained its vitality after spray drying, and its survival rate significantly improved following simulated digestion, heating, and environmental storage [119]. Salbutamol sulfate (SS) is a bronchodilator that acts as a short-acting β2-adrenergic agonist. It is commonly used to treat asthma and chronic obstructive pulmonary disease (COPD) [129]. The model drug SS is believed to exhibit improved encapsulation within gelatin A and gelatin B heteroprotein coacervation complexes. In vitro studies have shown that the release kinetics in simulated gastric juices follow a biphasic pattern [117].

### 4.2. Stabilizing Emulsion

Proteins, being amphiphilic molecules, possess hydrophilic and hydrophobic domains that can interact with water and oil, respectively. This dual interaction leads proteins to accumulate at the oil–water interface, creating a protective film around the oil droplets. Essentially, proteins envelop the oil droplet surface, resulting in the formation of microcapsules with an oil core and a protein membrane. The encapsulation of oil through oil-in-water emulsification techniques has widespread application in cosmetics, food, and pharmaceuticals. This process helps in diminishing the oily appearance of the product, facilitating controlled release of actives, reducing lipid oxidation, and preventing phase separation during storage [130]. In the field of food science, emulsions serve as a common vehicle for nutrient delivery, with proteins being particularly effective emulsifiers in Pickering emulsions [9]. Through composite coacervation, various proteins have the ability to self-assemble into nanoparticles, which can be utilized to stabilize Pickering emulsions.

TG is an enzyme that facilitates the deamidation and crosslinking reactions between proteins. The impact of oil gel on oil-gel-based Pickering emulsions was investigated, with TG-LF particles employed as the emulsifier [89]. The findings indicated that Pickering oil gel emulsions stabilized by TG-LF particles exhibited favorable curcumin release properties. In a separate investigation, an electrostatic complex was formed between LF and β-LG, leading to the neutralization of emulsion droplet charges at pH 7.0 [93]. Analysis of the droplet’s surface layer composition revealed that LF and β-LG proteins were adsorbed in equal proportions from the aqueous phase as complexes at pH 7.0. However, at pH 3.0, LF and β-LG proteins were observed to compete for adsorption. Specifically, at low protein concentrations (approximately 1%), β-LG protein was preferentially adsorbed, whereas at high protein concentrations, LF was preferentially adsorbed [93]. The sequential adsorption of WPIs and LF resulted in the formation of a double-layer nanoemulsion characterized by stable and relatively small droplets measuring 90 nm in diameter. Nanoemulsions stabilized through a double-layer structure comprising WPIs and LF exhibit greater stability compared to those formed solely by LF and WPIs, or a combination of both proteins, at pH 6 [94]. Ovotransferrin (OVT)–LYS particles with an OVT/LYS ratio of 8:1 at pH 9.3 fulfill all the criteria for effective Pickering stabilizers [9]. The Pickering emulsion demonstrated robust stability even after one month of storage at room temperature. At an oil content of 0.75, OVT-LYS particles exhibited an ability to stabilize Pickering emulsions with a high internal phase volume. The viscosity and gel-like consistency of Pickering emulsions were contingent upon the particle concentration and oil content. In comparison to the level of lipolysis observed in bulk oil, the lipolysis degree in Pickering emulsions stabilized by OVT-LYS particles increased by 39.4% [9]. Furthermore, encapsulating curcumin in OVT-LYS microparticle-stabilized Pickering emulsion enhanced the bioavailability of curcumin from 16.1% to 38.3%.

### 4.3. Protein Recovery and Extraction

At a specific pH value, two proteins with opposite charges can form complex coacervation or precipitation as a result of electrostatic interactions. Currently, there is a pressing need to create cost-effective and highly efficient technologies for protein purification that can be used on a large scale. Proteins exhibit minimal solubility when they are near their isoelectric pH, as they carry no net charge at this point. However, protein molecules possess an uneven charge distribution across various pH levels, leading to pH-dependent protein–protein interactions. This separation technology offers several advantages over other methods, including its simplicity, affordability, efficient use of solvents, and high speed. This phenomenon can be utilized in the recovery and extraction of proteins. LF coacervates with two subtypes of β-LG, namely β-LG A and β-LG B, consist of 162 amino acids and have molecular weights (Mws) of 18,362 Da and 18,277 Da, respectively. The two subtypes differ from each other in only two amino acids, specifically Asp 64 and Val 118. It is important to note that LF exhibits selective coagulation with the B-LG isotype A. This selectivity is demonstrated by the high yield (80%) of LF obtained when mixed with B-LG A over a wide range of concentrations, whereas the maximum yield achieved with B-LG B is 42% [95].

Bovine serum albumin (BSA) and LF underwent isoelectric separation using charged ultrafiltration membranes. The outcomes were utilized to identify the most effective conditions for protein separation. LF was fully retained in the feed mixture, while BSA, with a pI of 5.0 pH, yielded 30.31 m g^−2^ h^−1^ as the maximum permeation flux when an unmodified membrane was employed [96]. On the other hand, at pH 9.0 LF, BSA was entirely captured by the negatively charged membrane, resulting in LF achieving a maximum permeable flux of 1.07 m g^−2^ h^−1^. The yields of the β-CG/LYS complex at pH 6, 7, and 8 were 85.1%, 94.0%, and 97.3%, respectively [114]. With increasing pH, the greater number of binding sites between β-CG and LYS results in the displacement of more small ions and water by proteins, leading to an increase in the overlapping potential field. This shift favors precipitation over coacervation [114]. Another study demonstrated that β-LG did not affect BSA/GB coacervates, with only β-LG being separated from the BSA/β-LG/GB mixture during the coacervation process. The separation process involved β-LG being separated in the initial step, while BSA remained in the BSA-GB coacervation. Ethanol was then utilized to extract BSA from the BSA-GB coacervates. GB molecules tended to aggregate at the bottom, with BSA remaining in the supernatant obtained through centrifugation [118]. This further confirms that complex coagulation is an effective method for protein isolation and purification.

### 4.4. Nanohydrogel

Protein-based nanohydrogels have garnered significant interest for their non-toxic, biodegradable nature and small size, featuring large internal networks for multivalent bioconjugation. This characteristic allows for the encapsulation of functional components through covalent bonds [131,132]. Food protein nanohydrogels are stable networks formed by hydrophobic interactions, disulfide bonds, and hydrogen bonds through gelation. Heat-induced gelation unfolds the natural protein conformation, exposing functional groups such as sulfhydryl or hydrophobic groups, which then aggregate via disulfide bonds and hydrophobic interactions to minimize the system’s energy. Upon cooling, the formation of multiple hydrogen bonds significantly enhances elasticity [133]. Nanohydrogels were produced through the electrostatic interaction and thermal gelation of LF and GMP as primary materials. With increasing temperature, the aggregation level of LF and GMP blends rose, leading to a reduction in the fluid dynamic diameter and polydispersion index (PdI) of the particles. By combining LF and GMP solutions (0.02% (*w*/*w*), molar ratio 1:7) at pH 5.0, nanohydrogels can be obtained, followed by stirring and heating at 80 °C for 20 min. These findings indicate that the nanohydrogel exhibits a spherical shape with a hydrodynamic diameter of approximately 170 nm, a PdI of 0.1, and a swelling ratio of 30. These characteristics suggest that this system holds promise as a delivery mechanism for food and pharmaceutical applications [90]. In another study, a complex was formed through a heteroprotein interaction between OVA and LYS, with enhanced foaming and emulsifying capabilities at pH 2.0/4.0 [80]. A further pH increase improved the foam stability of the OVA-LYS complex. Moreover, within the pH range of 6.5–9.5, the gel network’s strength was bolstered by electrostatic interactions, hydrophobic interactions, SH-SS redox interconversion exchange, and hydrogen bonding. This leads to the formation of a viscous cohesive layer with excellent gel strength and water retention.

### 4.5. Infant Formula

Bioactive milk proteins play a crucial role in infant health and development. LF and osteopontin (OPN) are two multifunctional whey proteins found in human milk but not in cow milk. Human milk typically contains 1–7 g/L of LF, whereas cow milk only contains 0.03–0.1 g/L of LF [134,135]. To explore the morphology and comparable bioactivity of the milk LF-OPN complex in infant formula, research has been conducted. A 3:1 ratio of LF to OPN exhibited the most potent effects, indicating that the bovine LF-OPN complex, when combined with the formula protein, resisted in vitro digestion, promoted intestinal cell proliferation (15–50%) and differentiation (30–50%), enhanced antibacterial activity (25–50%), and boosted intestinal immunity [97]. Incorporating these proteins into infant formulas could lead to enhanced outcomes. In a separate study, among the four complexes formed by apo- and holo-LF/OPN, the apo-LF and holo-OPN complexes (AH) exhibited the most robust proliferative effects on human intestinal epithelial cells (HIECs) [98]. The AH complex demonstrated resistance to gastrointestinal digestion in vitro, colocalization with LF and OPN receptors, and the stimulation of HIEC proliferation through the activation of PI 3K/Akt signaling pathways. The formation of the LF-OPN complex could potentially aid both proteins in resisting digestion and enhance their capacity to support intestinal development in infants [98].

In summary, the applications of alkaline proteins in this section highlight their pivotal roles in enhancing the functional properties of food products, facilitating innovative extraction techniques, and providing health benefits, particularly in specialized areas such as infant nutrition. Continued exploration and optimization of these proteins and their interactions will further enhance their applicability and effectiveness in various industries, ultimately leading to improved health outcomes and product quality. Specifically, different basic proteins exhibit a variety of applications owing to their distinct structural properties and functional characteristics. For instance, LF, a glycoprotein with high iron-binding capacity, is widely utilized in complex coacervates. Its unique structure allows it to effectively embed active substances, making it an ideal choice for applications in nutraceuticals, dietary supplements, emulsifiers, and infant formulas to enhance nutrient absorption and offer antimicrobial benefits. On the other hand, LYS demonstrates exceptional performance in protein extraction processes. Its enzymatic activity is particularly beneficial in the food industry, where it is used to enhance protein recovery and increase the yield of various natural extracts. The structural stability of LYS also allows it to function effectively under a wide range of conditions, making it versatile for different extraction methodologies. Similarly, gelatin is renowned for its unique gelling properties and is prominently used in the formulation of nanohydrogels. Due to its biocompatibility and ability to form hydrogels upon hydration, gelatin is widely used in drug delivery systems, tissue engineering, and food technology. Its structural properties allow for the entrapment of bioactive compounds to enhance stability and to control release of pharmaceuticals.

## 5. Challenges and Approaches in the Application of HPCCs

### 5.1. Limitations in Delivery

**Figure 6:**
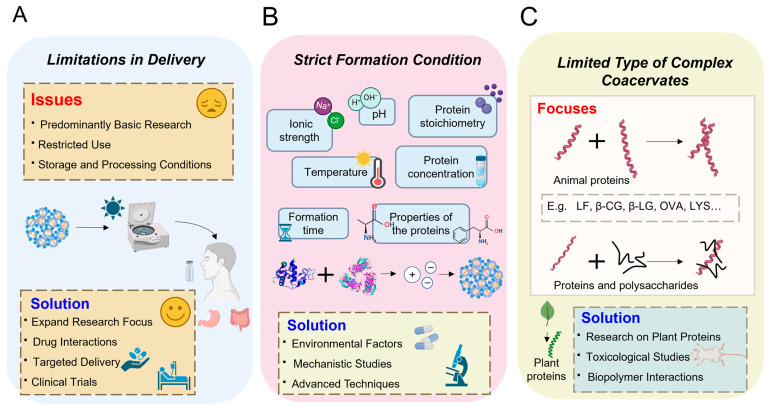
Challenges and approaches in the formation of heteroprotein complexes based on lactoferrin. (**A**) Limitations in delivery. (**B**) Strict formation condition. (**C**) Limited types of complex coacervates.

Currently, research on HPCCs in the context of the delivery of bioactive molecules and drugs primarily focuses on fundamental academic investigations. These studies provide valuable insights into the types used in preparing heteroprotein carriers and how these carriers affect the release properties of bioactive compounds. Despite these advancements, research on HPCCs as nanocarriers within delivery and drug delivery systems has thus far remained limited and incomplete (Figure 6A). For instance, questions persist regarding the suitability of HPCCs as a drug delivery vehicle, the specific interactions between different types of drugs and HPCCs, and the nuanced associations between HPCCs and the targeted organs or diseases. This lack of comprehensive understanding hinders the full exploration of HPCCs’ potential in advancing drug delivery systems. Further research is needed to elucidate the intricate mechanisms underlying the utilization of HPCCs in drug delivery and to unlock their full capabilities in enhancing therapeutic outcomes. The research gaps, concerning the digestion of HPCC materials within the gastrointestinal tract after incorporating active molecules, and their effects on the stability of storage and processing conditions, are currently underexplored. Understanding how HPCC materials are processed postingestion and their interactions within the digestive system is crucial for optimizing their effectiveness as drug delivery vehicles. Additionally, the insufficient exploration of how storage and processing conditions impact the stability and functionality of HPCCs presents an opportunity for further investigation. By delving deeper into these areas, researchers have the potential to enhance the design and advancement of HPCC-based drug delivery systems, ultimately resulting in more potent and efficient therapeutic interventions. A heightened focus on these aspects will pave the way for groundbreaking advancements in bioactive molecule and drug delivery technology, contributing to the field of food and pharmaceutical science.

### 5.2. Strict Formation Condition

Ther heteroprotein complex coacervation is characterized by spheroidal proteins with opposing charges and relies on a delicate equilibrium of short- and long-range Coulomb interactions among the proteins. Without this precise balance, the proteins may either aggregate into an amorphous precipitate or remain dispersed in the solution. Consequently, the formation of coacervates occurs within a highly restricted range of factors, including pH, ionic strength, protein concentration, and protein stoichiometry. The observation of coacervates within such narrow parameters underscores the sensitivity and specificity of the coacervation process. These conditions also highlight the intricate interplay between the charged proteins, emphasizing the critical role of electrostatic interactions in driving the formation of these coacervates (Figure 6B). Understanding and controlling these factors are essential for manipulating the coacervation process to achieve desired outcomes in drug delivery and other applications. Further exploration of the mechanisms governing heteroprotein complex coacervation will enhance our knowledge of these systems and facilitate the development of novel approaches for utilizing them in various food, biomedical, and pharmaceutical contexts.

### 5.3. Limited Types of Complex Coacervates

Current research on HPCC primarily centers around two animal proteins, because plant proteins tend to self-aggregate due to their inherent structure, resulting in a delicate balance of attractive and repulsive interactions. This inherent tendency of plant proteins to self-aggregate may pose challenges in their ability to form complex coacervates effectively. It is important to acknowledge that the exploration of complex coacervation dynamics and the analysis of the relationship between physical states and practical applications are still in their early stages. Given the limited research on the dynamics of complex coacervation systems within the food industry, the future trajectory of technology, theory, and research content is predominantly focused on the complex coacervation of biopolymers, encompassing proteins and polysaccharides. This focus extends beyond just the complex coacervation of heteroproteins, aiming to deepen the understanding of the interactions and behaviors of various biopolymers in complex coacervate systems. Moreover, insufficient toxicological studies on HPCC have created a significant gap in understanding its potential impact on food safety. The scarcity of biological scrutiny in the literature has raised concerns about the safety of utilizing HPCCs as carriers for drugs or bioactive compounds within the human body. To ensure the safe application of HPCCs, it is imperative to conduct thorough toxicological studies that assess the potential impact on human health. By addressing these concerns, researchers can enhance the understanding of the safety implications associated with HPCCs and pave the way for their responsible and effective utilization in various applications. By delving further into these areas, researchers can unlock new insights and potential applications for these complex systems in diverse fields (Figure 6C).

The critical discussions in this section reveal substantial limitations in the research and application of heteroprotein complex coacervates as delivery systems of bioactive compounds and drugs. While current studies provide foundational insights, the gaps in understanding the interactions, formation conditions, and safety of HPCCs hinder the translation into practical applications. By prioritizing comprehensive research efforts that address these challenges, the potential for HPCCs to revolutionize bioactive compound delivery technology can be realized. Future investigations to explore broader biopolymer interactions, optimize coacervation conditions, and assess safety implications will be crucial for advancing this innovative field and unlocking the diverse applications of HPCCs in food, nutrition, and materials science.

## 6. Conclusions and Outlooks

The focus of this review is to provide an overview of the latest advancements in research on the binding phase separation characteristics, potential mechanisms, and nutritional applications of heteroprotein coacervation. Additionally, it aims to offer a comprehensive summary of the challenges encountered in production practices. Understanding the biological function mechanisms of these heteroprotein complexes and their potential applications can pave the way for the development of innovative foods with specific functional traits, ultimately promoting their utilization in the food and nutritional industry. Despite the current infancy of research on heteroprotein complex coacervation, there is a pressing need for further exploration into the thermodynamics, kinetics, and binding models of complex coacervation. Furthermore, the application of heterogeneous protein composite coacervation in food products warrants further investigation, particularly in enhancing food functionalities such as foaming properties, membrane characteristics, gel properties, and the delivery of functional components or flavor additives. The next phase of research should shift focus towards exploring the practical applications of HPCCs rather than solely focusing on experimental basic research. Additionally, there is a need to optimize operating conditions to minimize process costs and environmental impact. This shift in focus will pave the way for the real-world implementation of HPCC technology in various sectors.

## Figures and Tables

**Table 3:** Properties, types, experimental index, and potential applications of heteroprotein complexes formed based on LYS.

Basic Protein	Interacting Protein	pI	Procedure to Prepare Heteroprotein Complexes	Application	References
LYS (pI~10)	SPI	-	Mass ratio SPI/LYS = 4:1–1:4, 20 mM NaCl	-	[79]
	OVA	4.5	A wide pH range with a 1:1 (OVA:LYS) mass ratio, the highest turbidity was observed at pH 7.5	-	[84]
	β-CG	-	pH 6 and 7 with 5–80 mM NaCl, pH 8 with 40–80 mM NaCl	-	[102]
	β-CG	-	40 mM NaCl, pH 8	-	[103]
	β-CG	-	Mass ratio β-CG/LYS = 1:1.3, pH = 6	Encapsulation of curcumin	[104]
	SPI	-	Mass ratios SPI/LYS = 2:1, 1:1.3, 200 mM NaCl	-	[105]
	β-casein	5.0	pH = 3–12, 0.15 M NaCl, temperature of 80 °C	-	[99]
	OVA	4.5	Mass ratio OVA:LYS increased from 10:0 to 10:10	-	[106]
	BSA	5.0	Mass ratio BSA:LYS = 1:2, pH = 9.0	-	[107]
	OVA	4.5	pH = 6.5–9.5	Gel network	[80]
	α-LA	4–5	Temperatures of 5–45 °C	-	[109]
	CAS	4.5	Mass ratio CAS:LYS = 1:1	-	[110]
	α-LA	-	Temperatures of 45 °C	-	[111]
	OVA	-	Molar ratio OVA/LYS = 2:3, temperature of 75 °C	-	[112]
	WPI	-	Mass ratio WPI/LYS = 1:2, pH = 7.5	Encapsulated for fucoxanthin	[113]
	OVT	-	Molar ratios OVT/LYS = 8:1, pH 9.3	Pickering emulsion for curcumin	[9]
	β-CG	-	pH 5.75–6.5	Increase in the overlapping potential field	[114]

“-” represents missing information or work not reported.

**Table 4:** Properties, types, experimental index, and potential applications of heteroprotein complexes formed based on gelatin.

Basic Protein	Interacting Protein	pI	Procedure to Prepare Heteroprotein Complexes	Application	References
Gelatin (pI~8)	BSA, β-LG	5.0	Mass ratio BSA/gelatin = 1, β-LG/gelatin = 0.75, BSA–gelatin/β-LG = 0.75	-	[85]
	Gelatin	-	Temperature of 5 °C	-	[108]
	LYS	-	LYS/gelatin weight ratios (0.01–100), temperatures of 18–40 °C, pH = 7	-	[116]
	Gelatin A and Gelatin B	9; 5	Gelatin A/gelatin B = 3:2, temperature of 37 °C	Encapsulation of a drug, salbutamol sulfate	[117]
	BSA, β-LG	5.0	Mass ratio BSA/gelatin > 1, Mass ratio β-LG/gelatin < 1	-	[118]
	CAS	-	Gelatin/CAS = 2, pH = 6.0	Encapsulate probiotics	[119]

“-” represents missing information or work not reported.

## Data Availability

No new data were created or analyzed in this study. Data sharing is not applicable to this article.
